# Optimization of the target strategy of perioperative infusion therapy based on monitoring data of central hemodynamics in order to prevent complications

**DOI:** 10.3389/fmed.2022.935331

**Published:** 2022-10-03

**Authors:** Dmytro Dmytriiev, Oleksandr Nazarchuk, Mykola Melnychenko, Bohdan Levchenko

**Affiliations:** ^1^Department of Anesthesiology and Intensive Care, National Pirogov Memorial Medical University, Vinnytsya, Ukraine; ^2^Department of Microbiology, National Pirogov Memorial Medical University, Vinnytsya, Ukraine

**Keywords:** ERAS (Enhanced Recovery After Surgery), GDT, infusion therapy, hemodynamic monitoring, central hemodynamics, cardiac output, esCCO, complications of infusion therapy

## Abstract

Enhanced Recovery After Surgery (ERAS) protocols are increasingly used in the perioperative period around the world. The concept of goal-directed fluid therapy (GDT) is a key element of the ERAS protocols. Inadequate perioperative infusion therapy can lead to a number of complications, including the development of an infectious process, namely surgical site infections, pneumonia, urinary tract infections. Optimal infusion therapy is difficult to achieve with standard parameters (e.g., heart rate, blood pressure, central venous pressure), so there are various methods of monitoring central hemodynamics – from invasive, minimally invasive to non-invasive. The latter are increasingly used in clinical practice. The current evidence base shows that perioperative management, specifically the use of GDT guided by real-time, continuous hemodynamic monitoring, helps clinicians maintain a patient’s optimal fluid balance. The manuscript presents the analytical data, which describe the benefits and basic principles of perioperative targeted infusion therapy based on central hemodynamic parameters to reduce the risk of complications.

## Introduction. Modern aspects of perioperative infusion therapy

Enhanced Recovery After Surgery (ERAS) protocols are increasingly used in the perioperative period around the world (1, 2). The introduction of ERAS protocols has reduced the hospital stay period by 30–50%, decreased the risk of complications and significantly reduced the frequency of re-hospitalizations (1–3). Goal-directed fluid therapy (GDT) is a key element of the ERAS protocols (4).

Monitoring of hemodynamics, volemia, blood loss, hemocoagulation and metabolism is the basis for selecting adequate methods to restore and maintain proper tissue perfusion. The value of monitoring lies in the use of the obtained data to determine the goals of therapeutic effects (5).

There is a list of clinical indications for circulatory optimization, the ultimate goal of which is the balance between delivery (DO_2_) and oxygen consumption (VO_2_). Indications may be due to the patient’s condition and the cause of circulatory failure (6):

– severe disease or damage to the cardiovascular and respiratory systems with severe functional disorders;
– age-related functional disorders of one or more organ systems;
– acute massive blood loss of traumatic and surgical origin (> 2.5l);
– severe sepsis;
– shock or severe hypovolemia of any origin;
– respiratory failure (PaO_2_ < 60 mm Hg, SaO_2_ < 90% in a patient on spontaneous breathing or PaO_2_/FiO_2_ < 300 mm Hg in a patient on mechanical ventilation);
– acute enteropathy (abdominal compartment syndrome, pancreatitis, perforation of internal organs, gastrointestinal bleeding);
– acute renal failure (urea > 20 mmol/l, creatinine > 200 μmol/l).
  In addition, there are indications associated with surgery:
– major non-cardiac surgery (pneumonectomy, resection of the liver, intestines, complex trauma and orthopedic interventions);
– major (combined) interventions on the heart and blood vessels (aortic aneurysm, combined prosthetic heart valves, coronary artery bypass grafting and carotid endarterectomy);
– long-term surgical interventions (for example, in neurosurgery, gastrointestinal surgery);
– urgent cavitary surgery (7).

Until recently, only invasive hemodynamic monitoring was possible to assess key indicators used in GDT protocols. However, in recent decades there have been restrictions on the use of pulmonary artery catheters (PAC) in the perioperative period. Routine use of a PAC is not recommended in patients with surgical pathology, except for heart surgery (8–10).

**FIGURE 1:**
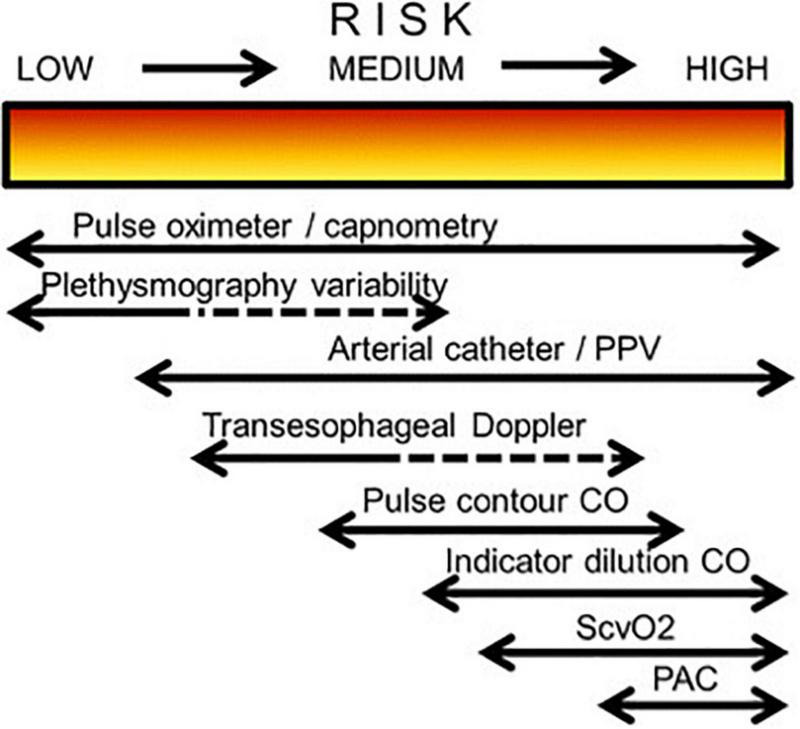
Possible choice of monitoring system in relation to a patient’s degree of perioperative risk (CO, cardiac output; PAC, pulmonary artery catheter; PPV, pulse pressure variation; ScvO_2_, central venous oxygen saturation).

The need for monitoring and its volume change over time, taking into account the patient’s condition, risk of complications, stage of the disease and intensive care (Figure 1) (11).

Perioperative infusion therapy plays an important role in reducing the risk of surgical infections. Both fluid overload and hypovolemia can impair tissue oxygenation, which adversely affects wound healing as well as the development of surgical infections (12, 13). Optimal infusion therapy is difficult to achieve with standard parameters [e.g., heart rate (HR), blood pressure (BP), central venous pressure (CVP)] (14). Surgical infections remain an important cause of morbidity and mortality in patients, ranking third in the incidence of healthcare associated infections. Surgical infections prolong the length of hospital stays, increase the cost of treatment and become a key indicator of the quality of care (15–17).

In recent years, various non-invasive hemodynamic monitoring technologies have been proposed (18). Innovative technologies for continuous non-invasive hemodynamic monitoring significantly expand the possibilities of improving the strategy of infusion therapy and personalization of hemodynamic management (19).

Among modern technologies of non-invasive monitoring, the pulse wave transit time (PWTT) is one of the newest ways to determine the main indicators of hemodynamics. The wide possibilities of this method is little studied. The strong correlation between stroke volume (SV) and PWTT discovered by Japanese scientists at Nihon Kohden was the basis of a formula that allows continuous monitoring of the most important volumetric parameters of the heart (stroke and heart indices). The results of a limited number of clinical trials of this non-invasive and convenient technique are available in the literature. An unequivocal opinion on this method has not yet been formed, but its accuracy and reliability for trend monitoring are considered quite satisfactory (20).

## Fluid management within Enhanced Recovery After Surgery protocols

**FIGURE 2:**
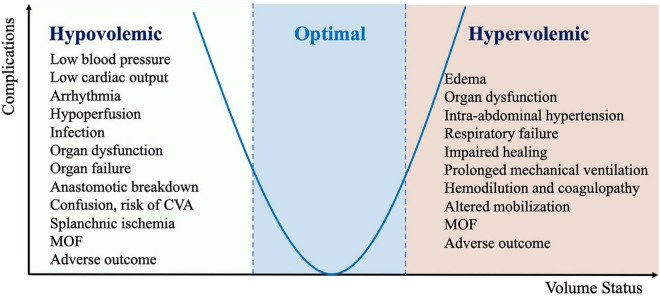
Complication associated with inadequate perioperative fluid management (CVA, cerebrovascular accident; MOF, multiple organ failure).

One critical element of all ERAS programs is a protocol known as perioperative goal-directed therapy (PGDT), which helps ensure adequate hydration and maintain euvolemia while avoiding hypervolemia or hypovolemia that can contribute to postoperative complications (Figure 2) (21, 22).

Fluid management within ERAS protocols should be viewed as a continuum through the preoperative, intraoperative, and postoperative period (1). The goal of preoperative fluid management is for the patient to be in a hydrated and euvolemic state when arriving in the operating room. This is usually achieved by avoidance of prolonged fasting and mechanical bowel preparation and encouraging patients to ingest a clear carbohydrate drink approximately 2 h prior to surgery. The goals of intraoperative fluid management are to avoid excess salt and water and to maintain central euvolemia. As such, patients undergoing surgery within an ERAS protocol should have an individualized fluid management plan. Maintenance of intravascular euvolemia throughout the perioperative period is ideal (23).

To achieve optimal fluid balance for the surgical patient, PGDT relies on continuous monitoring of a variety of hemodynamic targets, including cardiac output (CO), cardiac index (CI), stroke volume (SV), stroke volume variation (SVV), and pulse pressure variation (PPV).

Routine hemodynamic measurements, such as HR and mean arterial pressure (MAP), remain relatively unchanged despite reduced blood flow and are considered insensitive indicators of hypovolemia or changes in CI (24, 25). As a result, conventional fluid management is based on clinical assessment, vital signs, CVP monitoring, or a combination of these. However, recent studies have shown that CVP is not able to predict fluid responsiveness nor can changes in BP be used to approximate changes in SV or CO (26, 27).

Modern monitoring of perioperative infusion therapy is a personalized correction of hemodynamics, rather than on the basis of generally accepted schemes and rules. Some of the important requirements for monitoring methods are given in Table 1. The advanced hemodynamic monitoring equipment used to guide clinical decision-making intraoperatively be selected based on a combination of surgical patient and institutional factors (4).

**TABLE 1:** Requirements for the “ideal” method of monitoring.

Ensuring the measurement of the required indicators
Ensuring the accuracy and reproducibility of measurements
Obtaining data to be interpreted
Availability in clinical practice
Independence of results from operator skills
Fast response time
No risk of complications
Profitability
Providing the necessary information for the correction of treatment

## Methods of central hemodynamic monitoring of perioperative infusion therapy

**FIGURE 3:**
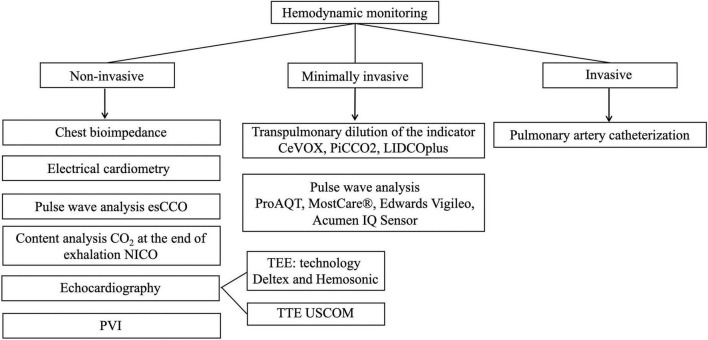
Methods of monitoring central hemodynamic (esCCO, estimated continuous cardiac output; NICO, non-invasive cardiac output partial CO_2_ rebreathing technique; PVI, pleth variability index; TEE, transesophageal echocardiography; TTE, transthoracic echocardiography, USCOM, ultrasonic cardiac output monitor; CeVOX, continuous central venous oxygenation measurement; PiCCO, pulse index continuous cardiac output; LiDCO, lithium dilution cardiac output).

Cardiac output monitoring is considered the gold standard for assessing central hemodynamic parameters and infusion response. There are many ways to measure CO, which differ in the degree of invasiveness and continuous or periodic research method (28–30). The various methods classification of monitoring central hemodynamics is presented in Figure 3.

### Invasive methods of hemodynamic monitoring

The classic invasive method is catheterization of the right heart with a Swan-Ganz catheter (Edwards Life Sciences, Irvine, CA, USA). Swan-Ganz advanced technology pulmonary artery catheters enable continuous assessment of flow, pressure, and oxygen delivery and consumption. Pulmonary artery catheterization or transpulmonary thermodilution is indicated for the most difficult patients to determine the type of shock. Swan-Ganz catheter allows continuous monitoring of the balance of oxygen delivery and consumption with the following advanced hemodynamic parameters: mixed venous oxygen saturation (SvO_2_), CO, SV, systemic vascular resistance (SVR), right ventricular ejection fraction (RVEF), right ventricular end diastolic volume (RVEDV), pulmonary artery pressure (PAP), pulmonary artery occlusion pressure (PAOP) (31).

This technique requires technical skills. Risks of a PAC procedure include arrhythmias, pneumothorax, heart block, lung infarction, perforation of the balloon, thrombosis, air embolism, knotting of the catheter, valvular damage, or infection (32). In less than 0.2% of cases Swan-Ganz catheterization results in serious vascular damage – pulmonary artery rupture (33).

To date, routine use of these methods is not recommended (34). Specific indications for PAC are cases of inconsistency of right ventricular and left ventricular (LV) contractility, pulmonary arterial hypertension, for example in cardiac surgery, lung and liver transplantation or severe ARDS (35–37). At the same time the CVP ceases to adequately reflect a preload of LV therefore for this purpose it is necessary to carry out monitoring of PAOP (38).

### Minimally invasive methods of hemodynamic monitoring

Minimally invasive methods based on usage transpulmonary dilution of the indicator (CeVOX, PiCCO_2_, LIDCOplus) and analysis of arterial pulse wave (ProAQT technology, MostCare^®^, Edwards Vigileo™ and Acumen IQ Sensor system).

The most established commercially available complete CeVOX and PiCCO_2_ sets are produced by Pulsion Medical Systems, Germany. The sensor for venous oximetry is installed through one of the lumens of the central venous catheter. CeVOX or PiCCO_2_ central units equipped with an optical module and a disposable fiber optic sensor are required for continuous measurement of venous saturation. There are studies that prove validity and clinical usefulness of the CeVOX (39–43). The PiCCO_2_ system allows continuous monitoring of blood oxygen supply and consumption values (44). Lithium dilution cardiac output (LiDCO™; LiDCO, London, UK) is a minimally invasive indicator dilution technique for the measurement of CO. The technique is quick and simple, requiring only an arterial line and central or peripheral venous access. A small dose of lithium chloride is injected as an intravenous bolus, and CO is derived from the dilution curve generated by a lithium-sensitive electrode attached to the arterial line.

ProAQT technology (Pulsion Medical Systems, Germany) is implemented as follows. The ProAQT sensor is an instantaneous pressure sensor and is inserted into a standard arterial catheter. A continuous blood pressure signal is supplied from the ProAQT sensor to a special monitor. Based on this signal, a number of hemodynamic parameters are determined. The most important thing in shock is the trend of minute blood volume (MBV). Initial calibration, which is calculated on the basis of basic anthropometric indicators (45), is required for continuous assessment of the MBV trend. There are other indicators that are determined by the ProAQT method, such as SVR and SVV.

MostCare^®^ (Vytech Health, Padua, Italy) is a method developed for continuous CO monitoring based on BP parameters. This technique does not require initial calibration and does not require central venous access. MostCare^®^ requires access to one of the peripheral arteries. The technology is based on the principle that a change in pressure changes the diameter of the vessel and, as a result, changes the volume of blood passing through its cross section. Variables such as LV contractility, pulse wave parameters, arterial wall elasticity, flexibility and peripheral resistance of small arterioles are closely interrelated and evaluated by the system. Thus, the flow gives the area under the curve, and then the formula is calculated by CO (46).

Edwards Vigileo™ system consists of a sensor (FloTrac, Edwards LLC) and a processing/display unit (Vigileo, Edwards LLC). The sensor is a transducer that preprocesses and sends a signal to both a cardiovascular monitor (for real time waveform display) and the Vigileo monitor. The processing unit applies a proprietary algorithm to the digitized wave, and reports CO, CI, SV, stroke volume index (SVI) and SVV. If a central venous pressure catheter has been placed, its signal can be interfaced with the Vigileo, allowing for the calculation of SVR and SVR index (SVRI). When used with a central venous oximetry catheter, the Vigileo also provides continuous central venous oxygen saturation (ScvO2).

Potential weaknesses of the system include possible inaccuracy in the presence of arterial wave artifact, compromise of the arterial catheter, aortic regurgitation, intense peripheral vasoconstriction and irregular pulse.

The Acumen IQ Sensor with Acumen hypotension prediction index (HPI) software (Edwards Lifesciences Ltd) is designed to predict the chance of an individual having a hypotensive event in surgical and non-surgical settings. A hypotensive event is defined as MAP of less than 65 mmHg, that exceeds a cumulative length of 15 min. The sensor attaches to any existing radial arterial line and is used to automatically calculate key parameters every 20 s. The parameters include HPI, contractility (systolic slope; dP/dt), afterload (dynamic arterial elastance), CO, pulse rate, SV, SVV, MAP, CI, PPV and SVR. HPI requires a good-quality arterial line waveform. To the extent that the waveform is damped or varies consequent to changes in patient position or other mechanical issues, predictions may be compromised.

### Non-invasive types of hemodynamic monitoring

Non-invasive monitoring is increasingly used in routine practice compared to invasive techniques, due to the lack of complications and the need for special technical skills, technical support (47).

Pleth variability index (PVi – volemia index; Masimo, Irvine, California, USA) – variations in the perfusion index during the respiratory cycle (Masimo Rainbow Pulse CO-Oximetry technology) (48). A number of independent objective studies have shown that Masimo SET technology provides the most reliable readings of oxygen saturation and HR measured in difficult clinical settings, including patient movements and low peripheral perfusion (49). PVi values are informative in predicting the response to fluid infusion in ventilated patients. However, changes in vasomotor tone, the appointment of vasopressors, hypothermia have a direct effect on plethysmographic signal and are potential limitations of the method.

The Non-invasive Cardiac Output partial CO_2_ rebreathing technique technology (NICO) introduced by Novametrix allows the measurement of CO by analyzing the CO_2_ content at the end of exhalation. The accuracy of NICO technology is lower compared to invasive techniques, also there is a dependence on ventilation and gas exchange. Partially reversible breathing is used to implement this technology. The monitor processor analyzes 4 parameters: the amount of carbon dioxide released during normal and reversible breathing and the content of carbon dioxide in the arterial blood during normal and reversible breathing (50). Specially conducted comparative studies of the results of CO registration by the NICO monitor and reference methods (Fick’s Principle, thermodilution) under critical conditions registered reliable correlation coefficients. Thus, it can be stated that this non-invasive method of CO registration is quite accurate (50).

ClearSight system finger cuff (Edwards Lifesciences) is a non-invasive device that is fixed on the patient’s finger. It allows continuous measurement of blood pressure. In addition, the device is able to calculate such parameters as CO, SVR, stroke volume, MAP.

The device has a so-called Heart Reference Sensor (HRS), which automatically compensates for changes in hydrostatic pressure due to differences in height between the finger and the heart. HRS compensates for changes in the patient’s arm position during any procedure or during patient movement (51).

Impedance cardiography (ICG) is a non-invasive method that uses changes in impedance of the chest to assess hemodynamic parameters, including CO. Studies of ICG have reported conflicting results and are difficult to compare, since they have been performed using devices of different generations in patients with different characteristics, while also using different equations (52). The technology is non-invasive, but the method is sensitive to electrical interference, patient movements, largely depending on the correct placement of electrodes. The accuracy of bioimpedance methods is questionable in a number of critical conditions (pulmonary edema, pleurisy, volume load, ventilation, arrhythmias, valve pathology) (50).

Electrical Cardiometry (EC) is a monitor for non-invasive method of measuring continuous CO monitoring based on measurement of thoracic electrical bioimpedance. Bioimpedance CO is based on the principle that cyclical increases in blood volume in the great vessels, as well as alignment of red blood cells (RBCs) in the thoracic aorta resulting from increased velocity, cause concomitant decreases in the electrical impedance in the chest. An alternating current of low amplitude is introduced and simultaneously sensed by electrodes placed around the neck, and laterally on the thorax to measure thoracic electrical bioimpedance. Changes in thoracic bioimpedance are induced by ventilation and pulsatile blood flow. EC is often confused with the traditional bioimpedance technology most commonly known as ICG. Though both methods use sensors placed on the thorax, traditional bioimpedance or ICG methods rely on the assumption of periodical volumetric changes in the aorta to determine SV and CO. ICG attributes the steep increase in the conductivity waveform to a volumetric expansion of the aorta during systole, while EC contributes the increase in conductivity to the orientation change of the RBCs to determine the velocity of the blood flow. EC method may be used for measuring cardiac output in a wide spectrum of diseases and patient populations including neonates and children, while ICG is limited to relatively healthy adults. The disadvantage associated with electric cardiometry is that the parameters are not available during electrical interference (electric cautery) (53).

The EC technology is utilized on the Aesculon and ICON device (Osypka Medical, Berlin, Germany/Cardiotronic, San Diego, CA, USA).

### Monitoring of hemodynamics by echocardiography

Echocardiography, as a non-invasive or semi-invasive method for the assessment of cardiac anatomy and function, is favored in clinical practice. The methods commonly used for echocardiography include transthoracic echocardiography (TTE), transesophageal echocardiography (TEE) and ultrasonic CO monitor (USCOM). TEE or TTE echocardiography can provide immediate point-of-care assessment of acute hemodynamic changes in selected patients. Indications for TEE include the evaluation of cardiac and aortic structure and function in situations in which the findings will alter management and the results of TTE are non-diagnostic or TTE is deferred because there is a high probability that results will be non-diagnostic. Situations in which TTE may be non-diagnostic include, but are not limited to, detailed evaluation of the abnormalities in structures that are typically in the far field, such as the aorta and the left atrial (LA) appendage; evaluation of prosthetic heart valves; evaluation of native valve masses; evaluation of paravalvular abscesses (both native and prosthetic valves); and various uses in critically ill patients. Transthoracic echocardiographic image quality may be compromised in patients on ventilators, those with chest wall injuries, obese patients, and those unable to move into the left lateral decubitus position.

Contraindications include esophageal disease with known stricture, diverticuli, varices or tumor, prior esophageal or stomach surgery, perforated viscus, or an uncooperative patient. Relative contraindications include cervical spine disease, hiatal hernia, coagulopathy, prior chest radiation, or facial or airway trauma. In addition to the estimation of CO (usually easier with TEE than with TTE), Doppler echocardiographic examination can provide an indication of cardiac function because it allows visualization of the cardiac chambers, valves, and pericardium (54). Doppler imaging can be used to calculate important indicators such as CO, CI, MBV, SVR, PAOP and a number of other indicators (55).

The USCOM ultrasonic cardiac output monitor (USCOMPty Ltd., Coffs Harbour, NSW, Australia) provides non-invasive transcutaneous measurement of CO. The flow profile is obtained by using a transducer (2.0 or 3.3 MHz) placed on the chest in either the left parasternal position to measure transpulmonary blood flow or the suprasternal position to measure transaortic blood flow. This flow profile is presented as a time–velocity spectral display showing variations of blood flow velocity with time. The CO is then calculated from the equation (56):

CO = heart rate × stroke volume

where the SV is the product of the velocity time integral (VTI) and the cross-sectional area (CSA) of the chosen valve.

The USCOM monitor is limited to cardiac output measurement and gives no indication of other hemodynamic variables, such as pressure measurements, vascular resistance or stroke work calculations. In addition, it does not provide the means to measure mixed venous oxygenation.

Ultrasonic transesophageal Doppler (Deltex, HemoSonic technologies) provide continuous assessment of CO by measuring the linear velocity of blood flow in the aorta. Doppler techniques are non-invasive and relative simple in application, but the results are approximate and depend on the position of the esophageal sensor and dysphagia may occur. Unstable hemodynamics and a narrow ultrasound window increase the measurement error. In addition, ultrasound techniques require a specially trained specialist (50).

CardioQ and CardioQ-ODM use Doppler ultrasound to monitor HR and intravascular fluid volume. CardioQ and CardioQ-ODM monitors are designed for use with a number of Doppler esophageal probes for Deltex Medical.

**FIGURE 4:**
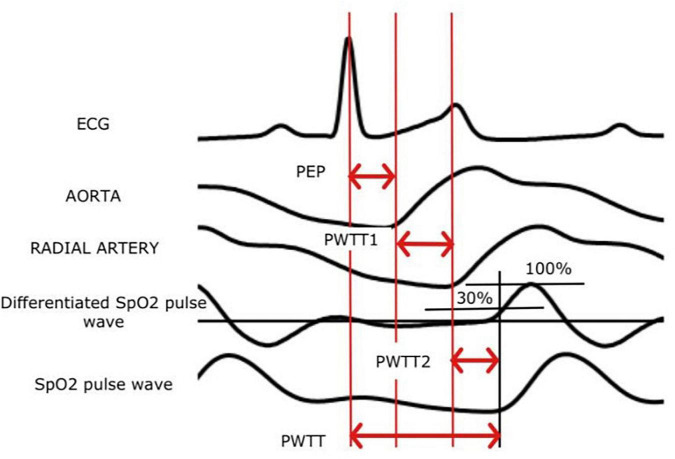
The principle of measuring esCCO (ECG, an electrocardiogram; PWTT, pulse wave transit time).

Recently, a non-invasive method of CO monitoring – estimated continuous cardiac output (esCCO), based on the assessment of pulse wave transit time (PWTT), has become available in clinical practice. The esCCO system defines PWTT as the time interval between the moment of appearance of the R wave on the electrocardiogram (ECG) and the beginning of the pulse wave on the plethysmogram of the pulse oximeter. This interval includes three points of measurement of time intervals (Figure 4): PEP – from R to ventricular contraction, T1 – the passage of a pulse wave from the aortic valve to the radial artery, T2 – from the radial artery to peripheral blood vessels. An inverse correlation was found between PWTT and SV (57–59).

Measured PWTT, BP and HR are used to calculate CO according to the following formula (60):

CO = K × (α × PWTT + β) × HR,

where CO – cardiac output; α is a constant that has been determined in previous clinical trials of esCCO technology; β is a variable that is a derivative of pulse pressure; K – calibration factor based on the biometric characteristics of the patient (height, weight, sex, and age); PWTT – pulse wave transit time; HR – heart rate.

Estimated continuous cardiac output technology is effective because it does not require to use of any additional sensors or specially trained personnel (61). EsCCO technology allows you continuously obtain indicators of central hemodynamics (CO, CI, SV, stroke index). Existing studies show that CO measured by esCCO and thermodilution (61, 62), showed a good correlation, with a small deviation (from 0.04 to 0.13 l/min). When comparing esCCO with TTE, the correlation was observed in cardiac patients with a range of −0.60 to 0.68 l/min (63), as well as in patients of intensive care unit with a deviation of −1.6 l/min (64).

However, the expressed disturbances of peripheral microcirculation, the presence of clinically significant cardiac arrhythmias, significant damage to peripheral arteries, severe heart valve dysfunction are the limitations of the method (65).

**FIGURE 5:**
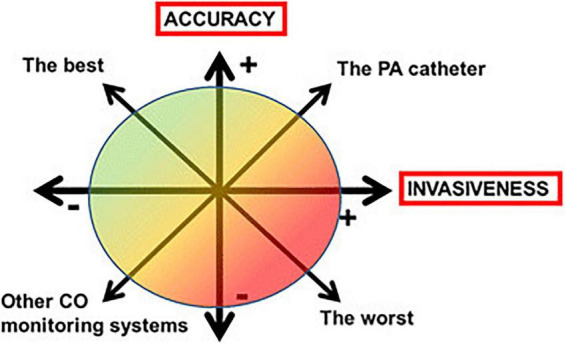
The compromise between accuracy and invasiveness of monitoring systems (CO, cardiac output; PA, pulmonary artery).

All monitoring systems have unique characteristics in terms of accuracy, reliability, measurement accuracy, stability and reliability (66). Physicians should take into account the technical limitations of each monitoring system and the potential compromise between more invasive but highly accurate CO measurements and less invasive but less accurate methods (Figure 5).

## Indications for different methods of cardiac output assessment during perioperative hemodynamic management

**FIGURE 6:**
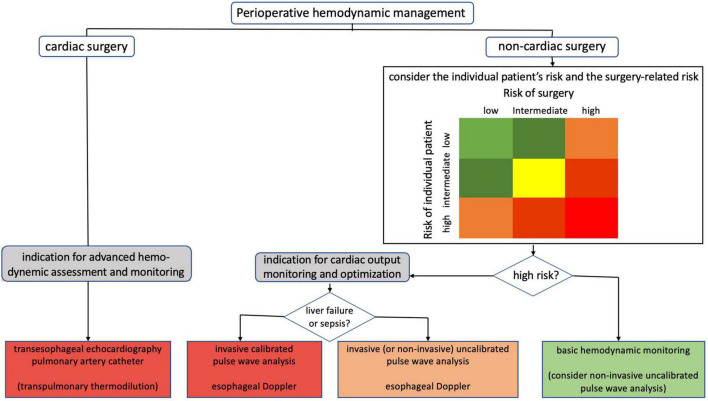
Indications for different methods of perioperative cardiac output assessment.

Indications for the different methods of CO assessment during the perioperative hemodynamic management of surgical patients are shown in Figure 6. In non-cardiac surgery patients, indications for CO monitoring depend on the presence of various patient-related and surgery-related risk factors for perioperative complications. The routine use of PAC to assess CO is not recommended (10). In addition, TEE is only recommended in patients with acute sustained severe hemodynamic instability in the perioperative period (10). Low-risk non-cardiac surgical patients can be monitored using basic hemodynamic monitoring (i.e., HR and rhythm, non-invasive arterial pressure, and peripheral oxygen saturation). In high-risk non-cardiac patients, monitoring of CO is indicated (11) as goal-directed hemodynamic management using fluids and inotropes to optimize CO (and oxygen delivery) has been shown to improve outcome (67–71). In high-risk non-cardiac surgical patients without marked alterations in vascular tone, invasive uncalibrated pulse wave analysis or esophageal Doppler can be used to guide CO optimization (71). Whether non-invasive uncalibrated pulse wave analysis can also be used for the assessment of CO in this category of patients is a subject of current research (72). In high-risk non-cardiac surgical patients with marked alterations in vascular tone (e.g., patients with liver failure or sepsis), CO can be assessed using invasive calibrated pulse wave analysis or esophageal Doppler (71). In patients undergoing open-heart and thoracic aortic surgery, TEE is indicated (73, 74). TEE may also be considered in coronary artery bypass graft surgery (73, 74). In selected cardiac surgery patients, advanced hemodynamic assessment and monitoring using a PAC may be considered.

## Traditional and modern assessment of response to infusion therapy

To assess the susceptibility to infusion, the simplest method is considered to be the test with passive raising of the legs by 30–45° to assess the response of CO and BP (75). Ventilation mode, type of fluid injected, starting position and measurement method do not affect the diagnostic efficiency of passive leg lift. It is considered the best test for fluid infusion response for patients with hypotension who do not require vasopressor therapy. Echocardiographic assessment of cardiac function is considered to be the best choice for more severe patients undergoing mechanical ventilation and vasopressor support. For patients in consciousness, spontaneous breathing, and with vasopressor support, a passive leg lift test is also recommended to assess the dynamics of changes in cardiac output (76).

**FIGURE 7:**
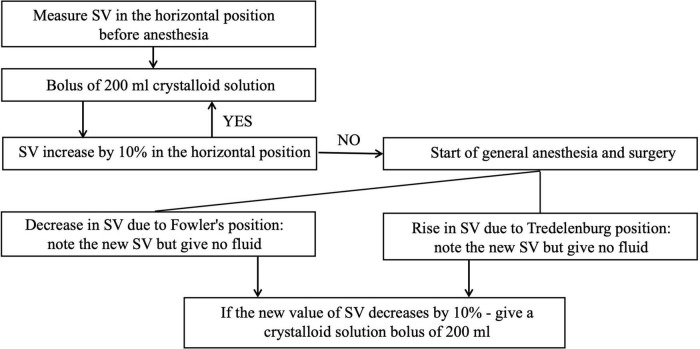
GDT protocol based on non-invasive monitoring of central hemodynamic (SV, stroke volume).

The response to infusion therapy using esCCO method can be assessed as follows. If infusion bolus of 500–1,000 ml caused a significant increase in cardiac output (4–6 l/min) and stroke volume (60–100 ml), the patient was considered susceptible to volemic therapy and continued to fill the circulating volume (77). In the absence of a positive hemodynamic response, the need for vasopressor and inotropic drugs was considered. It is available a GDT protocol recommended for the ultrasound method of hemodynamic monitoring (78), the modification of which can be used in other non-invasive monitoring techniques (Figure 7) (79).

## Complications with inadequate perioperative infusion therapy

Inadequate perioperative infusion therapy may lead to a decrease of CO and DO_2_ in damaged tissues, which is associated with an increased incidence of perioperative complications (Figure 2). In addition, the systemic inflammatory response associated with tissue damage leads to systemic capillary leakage syndrome and tissue edema. Limiting fluid intake and reducing diuresis may reduce edema in patients with reduced ventricular function, but increase the incidence of acute renal failure. There is increasing evidence from large databases that even short durations of hypotension with MAP < 65 mm Hg are associated with myocardial and kidney injury (80, 81).

Excessive infusion therapy can lead to a number of side effects, including coagulopathy and edema of the lungs, intestines, and peripheral tissues (11). Sodium and water retention after surgery may reduce the need for infusion. After stabilization of the patient’s condition, infusion therapy should be calculated only to restore the deficit and pathological losses.

The authors of multicenter observational study about clinical potential of an optimized perioperative fluid strategy in patients undergoing emergency gastrointestinal surgery (82) registered the postoperative complications as follows: wound-related complications included superficial wound rupture, rupture of the fascia, or anastomotic leakage. Cardiopulmonary complications included cardiac arrhythmia, acute myocardial infarction, cardiac arrest, pleural effusion, pulmonary congestion, pulmonary edema, congestive heart failure, or respiratory failure (failure to wean > 48 h, requiring continuous positive airway pressure after the day of extubating, or reintubation of any cause). Renal complications included the need for dialysis or other renal complications (nephritis or hydronephrosis treated with a nephrostomy catheter). Infectious complications included superficial wound infection, pneumonia, urinary tract infection, or cutaneous infection.

By improving cardiovascular function and balancing fluid intake, PGDT helps clinicians maintain adequate oxygen supply perioperatively. The implementation of PGDT protocols guided by continuous hemodynamic monitoring has also been shown to help decrease nausea, vomiting, and incidence of ileus (intestinal obstruction) while allowing patients to take solid food earlier, become more alert, and start walking sooner after surgery, ultimately reducing hospital LOS (21, 22).

The goal of targeted perioperative infusion therapy is to optimize the balance between delivery and oxygen consumption. Tissue hypoxia may occur with normal hemodynamic values such as MAP, CVP, and HR. Tissue hypoperfusion contributes to postoperative complications and increases the incidence of mortality, which requires adequate methods of enhanced hemodynamic monitoring and implementation of algorithms for targeted infusion therapy.

It is known that surgical patients following the principles of targeted infusion therapy during the perioperative period had a low risk of developing infectious complications, namely surgical site infections (SSIs), pneumonia and urinary tract infections (UTIs). There were no convincing data on catheter-related bloodstream infections (83).

Surgical patients carry a high overall risk of hospital-acquired infections (HAIs), mainly because SSIs occur in addition to non-surgery-specific infections (84). Despite existing prevention schemes, SSI remains one of the most common preventable surgical complications. SSIs occur in up to 20% of all abdominal surgeries and significantly contributing to morbidity, risk of death and increased treatment costs (85, 86). Surgical patients who did not receive surgical treatment also had a higher risk of infectious complications, including pneumonia and UTIs (84).

In surgical patients, the risk of infection is determined by the interaction between the microbes (degree of contamination and virulence), the patient (immune status) and the nature of the surgery (duration of the operation and the volume of damaged tissue). The infectious process occurs due to an unbalanced relationship between bacterial load and patient resistance. During the operation, the patient’s susceptibility to infection increases, the risk of infection increases due to damage to the integrity of the skin and mucous membranes, impaired microbicidal activity of immune cells (87). In this case, the perioperative delivery of oxygen DO_2_ plays an important role. It is known that a sufficient level of oxygen in the tissues promotes wound healing and increases resistance to infections (12, 88–90). This is because the oxidative function of neutrophils and the destruction of bacteria by alveolar macrophages (91) depend on adequate levels of oxygen in the tissue (88, 92). It is proved that low oxygen saturation in tissues is one of the prognostic factors in the development of SSIs (93). Therefore, maintaining adequate oxygen delivery to tissues is an important element in promoting a positive immune response to infection, especially in the context of surgery, which in itself leads to increased oxygen demand. Intraoperative increase in the ratio of oxygen in the inhaled air has not been shown to affect the postoperative wound and the risk of lung infections (94).

Oxygen delivery DO_2_ can be improved with GDT, which reduces the incidence of postoperative infectious complications. In the perioperative period, hypovolemia and decreased CO cause musculoskeletal and splanchnic vasoconstriction, which causes hypoperfusion and tissue hypoxia (95–97). This weakens the immune response of the mucous membrane and disrupts the intestinal barrier. Insufficiency of the intestinal barrier can lead to sepsis due to bacterial translocation and the release of cytokines into the blood, damaging other tissues and altering the body’s immune environment (98). In addition, ischemic reperfusion trauma of the intestine markedly disrupts intestinal lymphoid tissue (GALT), further weakens the immunity of the extraintestinal mucosa and contributes to the increased susceptibility of the patient to infections (98, 99).

Goal-directed fluid therapy aims to optimize DO_2_ by maintaining or increasing cardiac output. This preserves the microbicidal function of immune cells and the protection of organs that are particularly sensitive to perioperative hypoperfusion (21), avoiding intestinal barrier failure and GALT disorders. Adequate perioperative infusion therapy increases the oxygen content of tissues and increases the amount of collagen in wound healing (100).

Pathophysiological mechanisms of postoperative pneumonia are complex (101–103). Intestinal barrier failure and bacterial translocation through the lymphatic and thoracic ducts (104) with impaired airway mucosal immunity due to decreased DO_2_ have a potential pathogenetic role (105–107).

In surgical patients, GDT optimizes DO_2_ and prevents HAIs. Therefore, hemodynamically controlled principles of infusion therapy should be followed, especially in high-risk surgical patients (108–110) with a high probability of such complications.

The current evidence base shows that perioperative management, specifically the use of PGDT guided by real-time, continuous hemodynamic monitoring, helps clinicians maintain a patient’s optimal fluid balance. Meta-analyses of published studies focused on major abdominal surgery show that applying ERAS practice guidelines reduces postoperative complications by up to 50% and hospital LOS by 2.5 days (111, 112).

## Conclusion

Goal-directed fluid therapy is a key element of the ERAS protocols, which can only be achieved through high-quality monitoring. Among many techniques non-invasive hemodynamic monitoring is now evolving rapidly and is a highly accurate tool for clinical use. The use and further study of this method of hemodynamic monitoring can improve the understanding of the mechanisms underlying the systemic capillary leakage syndrome in critical conditions, and serve as a basis for developing new algorithms for GDT. Modern non-invasive technologies, like esCCO, allow to assess the heart failure as a component of circulatory failure and its targeted correction by optimizing the preload, postload and inotropic function of the heart, which meets most of the requirements for adequate continuous hemodynamic monitoring (7, 113).

Infusion therapy improves hemodynamic status by increasing SV and CO. CO changes of at least 10–15% are used to determine a positive response to fluid resuscitation after 250 to 500 ml of fluid infusion (33, 114, 115). If SV or CO increases, further fluids can be given in a controlled manner, repeating the fluid challenge so long as there is a positive response (SV maximization). This approach avoids fluid overload, as the only excess fluids are equivalent to one fluid challenge (116). Beyond immediate response to fluid infusion, the efficacy of a fluid bolus over time is affected by various parameters such as blood volume status, cardiac function, type of infused fluid, and capillary leak severity (96).

Goal-directed fluid therapy provides adequate systemic oxygenation, protects against ischemic-reperfusion injury and reduces the incidence of SSIs, respiratory infections and UTIs after surgery. Hemodynamically based perioperative optimization of infusion therapy reduces postoperative mortality and morbidity in surgical patients at high risk of infectious complications (67, 69, 70).

Therefore, it is necessary to continue research on aspects of perioperative GDT based on methods of monitoring central hemodynamics to reduce the risk of complications and improve the treatment outcomes of surgical patients.

## Author contributions

DD, ON, MM, and BL: investigation and resources. DD and MM: writing – original draft preparation. DD, ON, and MM: writing – review and editing. DD and ON: supervision. All authors have read and agreed to the published version of the manuscript.

## References

[1] Ljungqvist O Scott M Fearon KC. Enhanced recovery after surgery: a review. *JAMA Surg.* (2017) 152:292–8. 10.1001/jamasurg.2016.4952 28097305

[2] Eras Compliance Group. The impact of enhanced recovery protocol compliance on elective colorectal cancer resection: results from an international registry. *Ann Surg.* (2015) 261:1153–9. 10.1097/SLA.0000000000001029 25671587

[3] Savaridas T Serrano-Pedraza I Khan SK Martin K Malviya A Reed MR. Reduced medium-term mortality following primary total hip and knee arthroplasty with an enhanced recovery program. A study of 4,500 con-secutive procedures. *Acta Orthopaedica.* (2013) 84:40–3. 10.3109/17453674.2013.771298 23368747 PMC3584601

[4] Thiele RH Raghunathan K Brudney CS Lobo DN Martin D Senagore A American Society for Enhanced Recovery (ASER) and Perioperative Quality Initiative (POQI) joint consensus statement on perioperative fluid management within an enhanced recovery pathway for colorectal surgery. *Perioper Med (London, England).* (2016) 5:24. 10.1186/s13741-016-0049-9 27660701 PMC5027098

[5] Teylor BS Harbrecht BG Peitzman AB Rhodes Ì Schwab CW Ealy DM *The physiologic response to injury The Trauma Manual.* 2nd ed. Philadelphia, PA: Lippincott; Williams and Wilkins (2002). p. 17–20.

[6] Paromov KV Lenkin A I Kuzkov VV Kirov MY. Anaesthesiologist and hemodynamics: what the targeted therapy protocols will appear to result in? *Pacific Med J.* (2012) 3:17–21.

[7] Vincent JL Rhodes A Perel A Martin GS Della Rocca G Vallet B Clinical review: Update on hemodynamic monitoring-a consensus of 16. *Crit Care.* (2011) 15:229. 10.1186/cc10291 21884645 PMC3387592

[8] Seifi A Elliott RJ Elsehety MA. Usage of Swan-Ganz catheterization during the past 2 decades in United States. *J Crit Care.* (2016) 35:213–4. 10.1016/j.jcrc.2016.05.024 27325486

[9] Wiener RS Welch HG. Trends in the use of the pulmonary artery catheter in the United States, 1993-2004. *JAMA.* (2007) 298:423–9. 10.1001/jama.298.4.423 17652296

[10] Kristensen SD Knuuti J Saraste A Anker S Bøtker HE Hert SD 2014 ESC/ESA Guidelines on non-cardiac surgery: cardiovascular assessment and management: The Joint Task Force on non-cardiac surgery: cardiovascular assessment and management of the European Society of Cardiology (ESC) and the European Society of Anaesthesiology (ESA). *Eur Heart J.* (2014) 35:2383–431. 10.1093/eurheartj/ehu282 25086026

[11] Vincent JL Pelosi P Pearse R Payen D Perel A Hoeft A Perioperative cardiovascular monitoring of high-risk patients: a consensus of 12. *Critical care (London, England)* (2015) 19(1):224. 10.1186/s13054-015-0932-7 25953531 PMC4424585

[12] Jonsson K Jensen JA Goodson WH III Scheuenstuhl H West J Hopf HW Tissue oxygenation, anemia, and perfusion in relation to wound healing in surgical patients. *Ann Surg.* (1991) 214:605–13. 10.1097/00000658-199111000-00011 1953114 PMC1358617

[13] Hartmann M Jönsson K Zederfeldt B. Importance of dehydration in anastomotic and subcutaneous wound healing: an experimental study in rats. *Eur J Surg.* (1992) 158:79–82. 1350219

[14] Meregalli A Oliveira RP Friedman G. Occult hypoperfusion is associated with increased mortality in hemodynamically stable, high-risk, surgical patients. *Crit Care (London, England).* (2004) 8:R60–5. 10.1186/cc2423 15025779 PMC420024

[15] Kassavin DS Pascarella L Goldfarb MA. Surgical site infections: incidence and trends at a community teaching hospital. *Am J Surg.* (2011) 201:749–53. 10.1016/j.amjsurg.2010.03.002 21459358

[16] Leaper DJ van Goor H Reilly J Petrosillo N Geiss HK Torres AJ Surgical site infection - a European perspective of incidence and economic burden. *Int Wound J.* (2004) 1:247–73. 10.1111/j.1742-4801.2004.00067.x 16722874 PMC7951634

[17] Mulligan S Prentice J Scott L. *WoundsWest Wound Prevalence Survey 2011: State-wide Overview Report.* Perth, WA: Ambulatory Care Services, Department of Health (2011).

[18] Saugel B Cecconi M Wagner JY Reuter DA. Noninvasive continuous cardiac output monitoring in peri-operative and intensive care medicine. *Br J Anaesth.* (2015) 114:562–75. 10.1093/bja/aeu447 25596280

[19] Saugel B Vincent JL Wagner JY. Personalized hemodynamic management. *Curr Opin Crit Care.* (2017) 23:334–41. 10.1097/MCC.0000000000000422 28562384

[20] Terada T Maemura Y Yoshida K Samuna R Oiwa A Ochiai R. Comparison of estimated continuous cardiac output and transesophageal echocardiography cardiac output for noninvasively measuring cardiac output in pediatric patients undergoing kidney transplant surgery: 3AP4-2. *Eur J Anaesthesiol.* (2014) 31:39. 10.1186/s13054-016-1208-6 27885969 PMC5493079

[21] Giglio MT Marucci M Testini M Brienza N. Goal-directed haemodynamic therapy and gastrointestinal complications in major surgery: a meta-analysis of randomized controlled trials. *Br J Anaesth.* (2009) 103:637–46. 10.1093/bja/aep279 19837807

[22] Bellamy MC. Wet, dry or something else? *Br J Anaesth.* (2006) 97:755–7. 10.1093/bja/ael290 17098724

[23] Kendrick JB Kaye AD Tong Y Belani K Urman RD Hoffman C Goal-directed fluid therapy in the perioperative setting. *J Anaesthesiol Clin Pharmacol.* (2019) 35:S29–34. 10.4103/joacp.JOACP_26_18 31142956 PMC6515723

[24] Mythen MG Webb AR. Intraoperative gut mucosal hypoperfusion is associated with increased post-operative complications and cost. *Intensive Care Med.* (1994) 20:99–104. 10.1007/BF01707662 8201106

[25] Pierrakos C Velissaris D Scolletta S Heenen S De Backer D Vincent JL. Can changes in arterial pressure be used to detect changes in cardiac index during fluid challenge in patients with septic shock? *Intensive Care Med.* (2012) 38:422–8. 10.1007/s00134-011-2457-0 22278593

[26] Marik PE Baram M Vahid B. Does central venous pressure predict fluid responsiveness? A systematic review of the literature and the tale of seven mares. *Chest.* (2008) 134:172–8. 10.1378/chest.07-2331 18628220

[27] Le Manach Y Hofer CK Lehot JJ Vallet B Goarin JP Tavernier B Can changes in arterial pressure be used to detect changes in cardiac output during volume expansion in the perioperative period? *Anesthesiology.* (2012) 117:1165–74. 10.1097/ALN.0b013e318275561d 23135262

[28] Suehiro K Joosten A Alexander B Cannesson M. Guiding Goal-Directed Therapy. *Curr Anesthesiol Rep.* (2014) 4:360–75. 10.1007/s40140-014-0074-5

[29] Peeters Y Bernards J Mekeirele M Hoffmann B De Raes M Malbrain ML. Hemodynamic monitoring: To calibrate or not to calibrate? Part 1–Calibrated techniques. *Anaesthesiol Intensive Thera.* (2015) 47:487–500. 10.5603/AIT.a2015.0073 26578399

[30] Bernards J Mekeirele M Hoffmann B Peeters Y De Raes M Malbrain ML. Hemodynamic monitoring: To calibrate or not to calibrate? Part 2–Non-calibrated techniques. *Anaesthesiol Intensive Thera.* (2015) 47:501–16. 10.5603/AIT.a2015.0076 26578395

[31] Hensley FA Jr. Martin DE Gravely GP. *A Practical Approach to Cardiac Anesthesia.* Moscow: Medical news agency (2008).

[32] Labrunie E Levy C Paugam C Augereau B Tubiana JM. Pseudoanévrisme artériel pulmonaire post-cathéter de Swan-Ganz traité par embolisation [Pulmonary artery pseudoaneurysm caused by a Swan-Ganz catheter and treated by embolization]. *Ann Radiol.* (1993) 36:310–4. 8239472

[33] Stancofski ED Sardi A Conaway GL. Successful outcome in Swan-Ganz catheter-induced rupture of pulmonary artery. *Am Surg.* (1998) 64:1062–5. 9798769

[34] Cecconi M De Backer D Antonelli M Beale R Bakker J Hofer C Consensus on circulatory shock and hemodynamic monitoring. task force of the european society of intensive care medicine. *Intensive Care Med.* (2014) 40:1795–815. 10.1007/s00134-014-3525-z 25392034 PMC4239778

[35] Denault A Deschamps A Tardif JC Lambert J Perrault L. Pulmonary hypertension in cardiac surgery. *Curr Cardiol Rev.* (2010) 6:1–14. 10.2174/157340310790231671 21286273 PMC2845789

[36] Bozbas SS Bozbas H. Portopulmonary hypertension in liver transplant candidates. *World J Gastroenterol.* (2016) 22:2024–9. 10.3748/wjg.v22.i6.2024 26877607 PMC4726675

[37] Revercomb L Hanmandlu A Wareing N Akkanti B Karmouty-Quintana H. Mechanisms of pulmonary hypertension in Acute Respiratory Distress Syndrome (ARDS). *Front Mol Biosci.* (2021) 7:624093. 10.3389/fmolb.2020.624093 33537342 PMC7848216

[38] Lebedinskii KM. *Anesthesia and systemic hemodynamics (Assessment and correction of systemic hemodynamics during surgery and anesthesia).* Saint Petersburg: Chelovek (2000).

[39] Baulig W Dullenkopf A Hasenclever P Schmid ER Weiss M. *In vitro* evaluation of the CeVOX continuous central venous oxygenation monitoring system. *Anaesthesia.* (2008) 63:412–7. 10.1111/j.1365-2044.2007.05376.x 18336492

[40] Baulig W Dullenkopf A Kobler A Baulig B Roth HR Schmid ER. Accuracy of continuous central venous oxygen saturation monitoring in patients undergoing cardiac surgery. *J Clin Monit Comput.* (2008) 22:183–8. 10.1007/s10877-008-9123-2 18443743

[41] Müller M Löhr T Scholz S Thul J Akintürk H Hempelmann G. Continuous SvO2 measurement in infants undergoing congenital heart surgery–first clinical experiences with a new fiberoptic probe. *Paediatr Anaesth.* (2007) 17:51–5. 10.1111/j.1460-9592.2006.02026.x 17184432

[42] Huber D Osthaus WA Optenhöfel J Breymann T Marx G Piepenbrock S Continuous monitoring of central venous oxygen saturation in neonates and small infants: *in vitro* evaluation of two different oximetry catheters. *Paediatr Anaesth.* (2006) 16:1257–61. 10.1111/j.1460-9592.2006.01980.x 17121556

[43] Molnar Z Umgelter A Toth I Livingstone D Weyland A Sakka SG Continuous monitoring of ScvO(2) by a new fibre-optic technology compared with blood gas oximetry in critically ill patients: a multicentre study. *Intensive Care Med.* (2007) 33:1767–70. 10.1007/s00134-007-0743-7 17576533

[44] Michard F Perel A Management of circulatory and respiratory failure using less invasive volumetric and functional hemodynamic monitoring. In: Vincent JL Editor. *Yearbook of Intensive Care and Emergency Medicine*. New York, NY: Springer (2003). p. 508–20. 10.1007/978-1-4757-5548-0_48

[45] PROAQT. *Optimized Fluid Management*. (2020) Available online at: https://www.getinge.com/int/products/proaqt/ (accessed March 15, 2022).

[46] Romagnoli S Bevilacqua S Lazzeri C Ciappi F Dini D Pratesi C Most Care ^®^: a minimally invasive system for hemodynamic monitoring powered by the Pressure Recording Analytical Method (PRAM). *HSR Proc Intensive Care Cardiovasc Anesth.* (2009) 1:20–7. 23439735 PMC3484543

[47] Kirov MY Kuzkov VV. Optimization of hemodynamics in the perioperative period. *Bull Anesthesiol Resusc.* (2012) 5:56–66.

[48] Masimo. *Masimo Signal Extraction Technology.* (2020). Available online at: https://www.masimo.com/technology/co-oximetry/set/ (accessed September 15, 2020).

[49] Shah N Ragaswamy HB Govindugari K Estanol L. Performance of three new-generation pulse oximeters during motion and low perfusion in volunteers. *J Clin Anesth.* (2012) 24:385–91. 10.1016/j.jclinane.2011.10.012 22626683

[50] Malbrain M De Potter T Deeren D. Cost-effectiveness of minimally invasive hemodynamic monitoring. In: Vincent JL editor. *Yearbook of Intensive Care and Emergency Medicine.* New York, NY: Springer (2005). 10.1007/0-387-26272-5_52

[51] Edwards Lifesciences Corporation. *A noninvasive solution that enables clinical decision support to help optimize patient perfusion.* (2020). Available online at: https://www.edwards.com/gb/devices/Hemodynamic-Monitoring/clearsight (accessed September 15, 2020).

[52] Lorne E Mahjoub Y Diouf M Sleghem J Buchalet C Guinot PG Accuracy of impedance cardiography for evaluating trends in cardiac output: a comparison with oesophageal Doppler. *Br J Anaesth.* (2014) 113:596–602. 10.1093/bja/aeu136 24871872

[53] Rajput R Das S Chauhan S Bisoi A Vasdev S. Comparison of cardiac output measurement by noninvasive method with electrical cardiometry and invasive method with thermodilution technique in patients undergoing coronary artery bypass grafting. *World J Cardiovasc Surg.* (2014) 4:123–30. 10.4236/wjcs.2014.47019

[54] Repessé X Bodson L Vieillard-Baron A. Doppler echocardiography in shocked patients. *Curr Opin Crit Care.* (2013) 19:221–7. 10.1097/MCC.0b013e3283602344 23481099

[55] Mclean AS. Echocardiography in shock management. *Crit Care.* (2016) 20:275. 10.1186/s13054-016-1401-7 27543137 PMC4992302

[56] Tan H Parsons R Roberts B van Heerden P. Clinical evaluation of USCOM ultrasonic cardiac output monitor in cardiac surgical patients in intensive care unit. *Br J Anaesth.* (2005) 94:287–91. 10.1093/bja/aei054 15653709

[57] Romagnoli S Romano SM Payen D. Hemodinamics from the periphery. In: Vinsent J-L editor. *Annual Update in Intensive Care and Emergency Medicine 2011.* Berlin: Springer (2011). p. 424–33.

[58] Sugo Y Ukawa T Takeda S Ishihara H Kazama T Takeda J. A novel continuous cardiac output monitor based on pulse wave transit time. *Annu Int Conf IEEE Eng Med Biol Soc.* (2010) 2010:2853–6. 10.1109/IEMBS.2010.5626343 21095971

[59] Yamada T Sugo Y Takeda J ResearchTeam, esCCO. Verification of a non-invasive continuous cardiac output measurement method based on the pulse-contour analysis combined with pulse wave transit time: 3AP5–9. *Eur J Anaesthesiol*. (2010) 27:57. 10.1097/00003643-201006121-00179

[60] Gosling RG Budge MM. Terminology for describing the elastic behavior of arteries. *Hypertension.* (2003) 41:1180–2. 10.1161/01.HYP.0000072271.36866.2A 12756217

[61] Yamada T Tsutsui M Sugo Y Sato T Akazawa T Sato N Multicenter study verifying a method of noninvasive continuous cardiac output measurement using pulse wave transit time: a comparison with intermittent bolus thermodilution cardiac output. *Anesth Analg.* (2012) 115:82–7. 10.1213/ANE.0b013e31824e2b6c 22467885

[62] Tsutsui M Araki Y Masui K Kazama T Sugo Y Archer TL Pulse wave transit time measurements of cardiac output in patients undergoing partial hepatectomy: a comparison of the esCCO system with thermodilution. *Anesth Analg.* (2013) 117:1307–12. 10.1213/ANE.0b013e3182a44c87 24257379

[63] Mansencal N Delobelle J Balagny P Badie J Ihaddaden M Arslan M Usefulness of a noninvasive cardiac output measurement using pulse wave transit time in coronary care unit. *Int J Cardiol.* (2013) 168:4411–2. 10.1016/j.ijcard.2013.05.032 23714596

[64] Bataille B Bertuit M Mora M Mazerolles M Cocquet P Masson B Comparison of esCCO and transthoracic echocardiography for non-invasive measurement of cardiac output intensive care. *Br J Anaesth.* (2012) 109:879–86. 10.1093/bja/aes298 22907340

[65] Feissel M Aho LS Georgiev S Tapponnier R Badie J Bruyère R Pulse wave transit time measurements of cardiac output in septic shock patients: a comparison of the estimated continuous cardiac output system with transthoracic echocardiography. *PLoS One.* (2015) 10:e0130489. 10.1371/journal.pone.0130489 26126112 PMC4488420

[66] Thiele RH Bartels K Gan TJ. Cardiac output monitoring: a contemporary assessment and review. *Crit Care Med.* (2015) 43:177–85. 10.1097/CCM.0000000000000608 25251758

[67] Cecconi M Corredor C Arulkumaran N Abuella G Ball J Grounds RM Clinical review: Goal-directed therapy-what is the evidence in surgical patients? The effect on different risk groups. *Crit Care (London, England).* (2013) 17:209. 10.1186/cc11823 23672779 PMC3679445

[68] Pearse RM Harrison DA MacDonald N Gillies MA Blunt M Ackland G Effect of a perioperative, cardiac output-guided hemodynamic therapy algorithm on outcomes following major gastrointestinal surgery: a randomized clinical trial and systematic review. *JAMA.* (2014) 311:2181–90. 10.1001/jama.2014.5305 24842135

[69] Gurgel ST do Nascimento P Jr. Maintaining tissue perfusion in high-risk surgical patients: a systematic review of randomized clinical trials. *Anesth Analg.* (2011) 112:1384–91. 10.1213/ANE.0b013e3182055384 21156979

[70] Hamilton MA Cecconi M Rhodes A. A systematic review and meta-analysis on the use of preemptive hemodynamic intervention to improve postoperative outcomes in moderate and high-risk surgical patients. *Anesth Analg.* (2011) 112:1392–402. 10.1213/ANE.0b013e3181eeaae5 20966436

[71] Singer M. Oesophageal Doppler monitoring: should it be routine for high-risk surgical patients? *Curr Opin Anaesthesiol.* (2011) 24:171–6. 10.1097/ACO.0b013e32834452b2 21293263

[72] Nicklas JY Saugel B. Non-Invasive hemodynamic monitoring for hemodynamic management in perioperative medicine. *Front Med.* (2017) 4:209. 10.3389/fmed.2017.00209 29218310 PMC5703831

[73] American Society of Anesthesiologists and Society of Cardiovascular Anesthesiologists Task Force on Transesophageal Echocardiography. Practice guidelines for perioperative transesophageal echocardiography. An updated report by the American Society of Anesthesiologists and the Society of Cardiovascular Anesthesiologists Task Force on Transesophageal Echocardiography. *Anesthesiology.* (2010) 112:1084–96. 10.1097/ALN.0b013e3181c51e90 20418689

[74] Hahn RT Abraham T Adams MS Bruce CJ Glas KE Lang RM Guidelines for performing a comprehensive transesophageal echocardiographic examination: Recommendations from the American Society of Echocardiography and the Society of Cardiovascular Anesthesiologists. *Anesth Analg.* (2014) 118:21–68. 10.1213/ANE.0000000000000016 24356157

[75] Cherpanath TG Hirsch A Geerts BF Lagrand WK Leeflang MM Schultz MJ Predicting fluid responsiveness by passive leg raising: A systematic review and meta-analysis of 23 clinical trials. *Crit Care Med.* (2016) 44:981–91. 10.1097/CCM.0000000000001556 26741579

[76] Boyd JH Sirounis D. Assessment of adequacy of volume resuscitation. *Curr Opin Crit Care.* (2016) 22:424–7. 10.1097/MCC.0000000000000344 27478966

[77] Self WH Semler MW Wanderer JP Wang L Byrne DW Collins SP Balanced crystalloids versus Saline in Noncritically ill adults. *N Engl J Med.* (2018) 378:819–28. 10.1056/NEJMoa1711586 29485926 PMC5846618

[78] Brandstrup B Svendsen PE Rasmussen M Belhage B Rodt SÅ Hansen B Which goal for fluid therapy during colorectal surgery is followed by the best outcome: Near-maximal stroke volume or zero fluid balance? *Br J Anaesth.* (2012) 109:191–9. 10.1093/bja/aes163 22710266

[79] Volkov PA Sevalkin SA Churadze BT Volkova YT Gur’yanov VA. Goal-target infusion therapy based on noninvasive hemodynamic monitoring esCCO. *Anesteziol Reanimatol.* (2015) 60:19–23. 26596026

[80] Salmasi V Maheshwari K Yang D Mascha EJ Singh A Sessler D I Relationship between intraoperative hypotension, defined by either reduction from baseline or absolute thresholds, and acute kidney and myocardial injury after noncardiac surgery: A retrospective cohort analysis. *Anesthesiology.* (2017) 126:47–65. 10.1097/ALN.0000000000001432 27792044

[81] Maheshwari A McCormick PJ Sessler DI Reich DL You J Mascha EJ Prolonged concurrent hypotension and low bispectral index (‘double low’) are associated with mortality, serious complications, and prolonged hospitalization after cardiac surgery. *Br J Anaesth.* (2017) 119:40–9. 10.1093/bja/aex095 28974062 PMC6172972

[82] Voldby AW Aaen AA Loprete R Eskandarani HA Boolsen AW Jonck S Perioperative fluid administration and complications in emergency gastrointestinal surgery—an observational study. *Perioper Med.* (2022) 11:9. 10.1186/s13741-021-00235-y 35189974 PMC8862386

[83] Dalfino L Giglio MT Puntillo F Marucci M Brienza N. Haemodynamic goal-directed therapy and postoperative infections: earlier is better. A systematic review and meta-analysis. *Crit Care (London, England).* (2011) 15:R154. 10.1186/cc10284 21702945 PMC3219028

[84] Sax H Uçkay I Balmelli C Bernasconi E Boubaker K Mühlemann K Overall burden of healthcare-associated infections among surgical patients. Results of a national study. *Ann surg.* (2011) 253:365–70. 10.1097/SLA.0b013e318202fda9 21217517

[85] Kurz A Sessler DI Lenhardt R. Perioperative normothermia to reduce the incidence of surgical-wound infection and shorten hospitalization. Study of Wound Infection and Temperature Group. *N Engl J Med.* (1996) 334:1209–15. 10.1056/NEJM199605093341901 8606715

[86] Haley RW Culver DH Morgan WM White JW Emori TG. Identifying patients at high risk of surgical wound infection. A simple multivariate index of patient susceptibility and wound contamination. *Am J Epidemiol.* (1985) 121:206–15. 10.1093/oxfordjournals.aje.a113991 4014116

[87] El-Maallem H Fletcher J. Effects of surgery on neutrophil granulocyte function. *Infect Immun.* (1981) 32:38–41. 10.1128/iai.32.1.38-41.1981 6260683 PMC350583

[88] Hopf HW Hunt TK West JM Blomquist P Goodson WH III Jensen JA Wound tissue oxygen tension predicts the risk of wound infection in surgical patients. *Arch surg.* (1997) 132:997–1005. 10.1001/archsurg.1997.01430330063010 9301613

[89] Hohn DC MacKay RD Halliday B Hunt TK. Effect of O2 tension on microbicidal function of leuko-cytes in wounds and *in vitro*. *Surg Forum.* (1976) 27:18–20. 1019847

[90] Knighton DR Fiegel VD Halverson T Schneider S Brown T Wells CL. Oxygen as an antibiotic. The effect of inspired oxygen on bacterial clearance. *Arch Surg.* (1990) 125:97–100. 10.1001/archsurg.1990.01410130103015 2403785

[91] Allen DB Maguire JJ Mahdavian M Wicke C Marcocci L Scheuenstuhl H Wound hypoxia and acidosis limit neutrophil bacterial killing mechanisms. *Arch Surg.* (1997) 132:991–6. 10.1001/archsurg.1997.01430330057009 9301612

[92] Babior BM. Oxygen-dependent microbial killing by phagocytes (first of two parts). *N Engl J Med.* (1978) 298:659–68. 10.1056/NEJM197803232981205 24176

[93] Govinda R Kasuya Y Bala E Mahboobi R Devarajan J Sessler DI Early postoperative sub-cutaneous tissue oxygen predicts surgical site infection. *Anesth Analg.* (2010) 111:946–52. 10.1213/ANE.0b013e3181e80a94 20601453

[94] Meyhoff CS Wetterslev J Jorgensen LN Henneberg SW Høgdall C Lundvall L Effect of high perioperative oxygen fraction on surgical site infection and pulmonary complications after abdominal surgery: the PROXI randomized clinical trial. *JAMA.* (2009) 302:1543–50. 10.1001/jama.2009.1452 19826023

[95] Pessaux P Msika S Atalla D Hay JM Flamant Y French Association for Surgical Research. Risk factors for postoperative infectious complications in noncolorectal abdominal surgery: a multivariate analysis based on a prospective multicenter study of 4718 patients. *Arch Surg.* (2003) 138:314–24. 10.1001/archsurg.138.3.314 12611581

[96] Chappell D Jacob M Hofmann-Kiefer K Conzen P Rehm M. A rational approach to perioperative fluid management. *Anesthesiology.* (2008) 109:723–40. 18813052 10.1097/ALN.0b013e3181863117

[97] Miles AA Miles EM Burke J. The value and duration of defence reactions of the skin to the primary lodgement of bacteria. *Br J Exp Pathol.* (1957) 38:79–96. 13413084 PMC2082171

[98] Holland J Carey M Hughes N Sweeney K Byrne PJ Healy M Intraoperative splanchnic hypoperfusion, increased intestinal permeability, down-regulation of monocyte class II major histocompatibility complex expression, exaggerated acute phase response, and sepsis. *Am J Surg.* (2005) 190:393–400. 10.1016/j.amjsurg.2005.03.038 16105525

[99] Clark JA Coopersmith CM. Intestinal crosstalk: a new paradigm for understanding the gut as the “motor” of critical illness. *Shock (Augusta, Ga.).* (2007) 28:384–93. 10.1097/shk.0b013e31805569df 17577136 PMC2084394

[100] Hartmann M Jönsson K Zederfeldt B. Effect of tissue perfusion and oxygenation on accumulation of collagen in healing wounds. Randomized study in patients after major abdominal operations. *Eur J Surg.* (1992) 158:521–6. 1360822

[101] Kotani N Takahashi S Sessler DI Hashiba E Kubota T Hashimoto H Volatile anesthetics augment expression of proinflammatory cytokines in rat alveolar macrophages during mechanical ventilation. *Anesthesiology.* (1999) 91:187–97. 10.1097/00000542-199907000-00027 10422944

[102] Kotani N Lin CY Wang JS Gurley JM Tolin FP Michelassi F Loss of alveolar macrophages during anesthesia and operation in humans. *Anesth Analg.* (1995) 81:1255–62. 10.1097/00000539-199512000-00023 7486113

[103] Kotani N Hashimoto H Sessler DI Kikuchi A Suzuki A Takahashi S Intraoperative modulation of alveolar macrophage function during isoflurane and propofol anesthesia. *Anesthesiology.* (1998) 89:1125–32. 10.1097/00000542-199811000-00012 9822000

[104] Kusano C Baba M Takao S Sane S Shimada M Shirao K Oxygen delivery as a factor in the development of fatal postoperative complications after oesophagectomy. *Br J Surg.* (1997) 84:252–7. 9052449

[105] Kudsk KA Li J Renegar KB. Loss of upper respiratory tract immunity with parenteral feeding. *Ann Surg.* (1996) 223:629–38. 10.1097/00000658-199606000-00001 8645036 PMC1235201

[106] Fukatsu K Sakamoto S Hara E Ueno C Maeshima Y Matsumoto I Gut ischemia-reperfusion affects gut mucosal immunity: a possible mechanism for infectious complications after severe surgical insults. *Crit Care Med.* (2006) 34:182–7. 10.1097/01.ccm.0000196207.86570.16 16374173

[107] Pearse RM Harrison DA James P Watson D Hinds C Rhodes A Identification and characterisation of the high-risk surgical population in the United Kingdom. *Crit Care (London, England).* (2006) 10:R81. 10.1186/cc4928 16749940 PMC1550954

[108] Boyd O Jackson N. Clinical review: How is risk defined in high-risk surgical patient management? *Crit Care.* (2005) 9:390. 10.1186/cc3057 16137389 PMC1269426

[109] Lees N Hamilton M Rhodes A. Clinical review: Goal-directed therapy in high risk surgical patients. *Crit Care (London, England).* (2009) 13:231. 10.1186/cc8039 19863764 PMC2784362

[110] Corcoran T Rhodes JE Clarke S Myles PS Ho KM. Perioperative fluid management strategies in major surgery: a stratified meta-analysis. *Anesth Analg.* (2012) 114:640–51. 10.1213/ANE.0b013e318240d6eb 22253274

[111] Varadhan KK Neal KR Dejong CH Fearon KC Ljungqvist O Lobo DN. The enhanced recovery after surgery (ERAS) pathway for patients undergoing major elective open colorectal surgery: a meta-analysis of randomized controlled trials. *Clin Nutr (Edinburgh, Scotland).* (2010) 29:434–40. 10.1016/j.clnu.2010.01.004 20116145

[112] Marx G Schindler AW Mosch C Albers J Bauer M Gnass I Intravascular volume therapy in adults: guidelines from the association of the scientific medical societies in Germany. *Eur J Anaesthesiol.* (2016) 33:488–521. 10.1097/EJA.0000000000000447 27043493 PMC4890839

[113] Shere-Wolfe RF Galvagno SM Jr. Grissom TE. Critical care considerations in the management of the trauma patient following initial resuscitation. *Scand J Trauma Resusc Emerg Med.* (2012) 20:68. 10.1186/1757-7241-20-68 22989116 PMC3566961

[114] Vincent JL Weil MH. Fluid challenge revisited. *Crit Care Med.* (2006) 34:1333–7. 10.1097/01.CCM.0000214677.76535.A5 16557164

[115] Cecconi M Parsons AK Rhodes A. What is a fluid challenge? *Curr Opin Crit Care.* (2011) 17:290–5. 10.1097/MCC.0b013e32834699cd 21508838

[116] Fiddian-Green RG Baker S. Nosocomial pneumonia in the critically ill: product of aspiration or translocation? *Crit Care Med.* (1991) 19:763–9. 2055052

